# Prevalence and correlates of prior experimentation with e-cigarettes over conventional cigarettes among adolescents: Findings from the 2015 Korea Youth Risk Behaviour Web-based Survey

**DOI:** 10.18332/tpc/112595

**Published:** 2019-10-07

**Authors:** Jung Hyeon Hyeon, Cameron Shelley, Cheol Min Lee

**Affiliations:** 1 Department of Family Medicine, BHS Hanseo Hospital, Busan, South Korea; 2 Cancer Epidemiology Intelligence Division, Cancer Council Victoria, Melbourne, Australia; 3 Healthcare System Gangnam Center, Seoul National University Hospital, Seoul, South Korea

**Keywords:** smoking, public health, cross-sectional studies, electronic cigarette

## Abstract

**INTRODUCTION:**

As concern is increasing about electronic cigarette use among never-smoking youth, we aimed to examine the prevalence and correlates of prior experimentation of electronic cigarettes (e-cigs) over conventional cigarettes (c-cigs).

**METHODS:**

We used the 10th Korea Youth Risk Behavior Web-based Survey in 2015, including 67960 participants as study subjects. This survey was designed as stratified multistage clustered samples from middle schools and high schools. Weighted percentages of vaping and/or smoking status by the timing of experimentation were calculated and multivariate logistic regression analysis was conducted after adjustments for possible confounders (demographics, socioeconomic status, lifestyle, tobacco use pattern).

**RESULTS:**

Youth who use e-cigs only or before c-cigs were 1.7% and 9.1% of any type user, respectively. In younger participants, the proportion tended to be increasing. Apart from being younger (AOR=2.23, 95% CI: 1.66–2.99; 12th grade vs 7th grade), male gender (AOR=1.20, 95% CI: 1.03–1.42), higher household income (AOR=1.21, 95% CI: 1.01–1.45), higher school performance (AOR=1.19, 95% CI: 1.02–1.39), exposure to smoke (AOR=1.63, 95% CI: 1.43–1.86) and caffeine drink (AOR=1.44, 95% CI: 1.24–1.68) were associated with experimentation with e-cigs prior to c-cigs in a fully-adjusted model. Alcohol abuse (AOR=0.57, 95% CI: 0.48–0.68) and weekday internet usage for recreation (AOR=0.69, 95% CI: 0.60–0.78) were negatively associated.

**CONCLUSIONS:**

The characteristics of those who experiment with e-cigs over c-cigs may be different from the general characteristics of vaping. Considering recent e-cig epidemics, more attention should be paid to the adolescents who tend to start e-cigs first.

## INTRODUCTION

The electronic cigarette (e-cig) is a battery-powered device that heats a solution, usually containing nicotine and various flavors, to be inhaled by the user^1^. Since its first appearance in the market, the e-cig has not only gained tremendous worldwide attention and popularity, but also has brought about numerous controversies and debate across the globe.

E-cig use has been increasing in the past decades, and it is still growing among the adult and adolescent population. In the US, self-reported e-cig usage among high school students has increased from 1.5% to 20.8%, and from 0.6% to 4.9% among middle school students (2011–2018)^2^. This trend was more evident in 2014 when the prevalence of e-cigs surpassed that of conventional cigarettes (c-cigs) among the youth population^3^. The number of Korean adolescents with e-cig experience since the introduction of e-cigs to Korea in 2007 also rose from 0.5% to 9.4% with an increase of 76.7% (estimated) in the dual-user population (2008– 2011)^4^.

With the surge in e-cig users, the appearances of e-cigs and brand marketing have become more dominant, particularly in movies, television shows, and the media. However, this social spotlight has rather resulted in negative implications, as this novel form of excessive marketing succeeded in establishing a distorted image of e-cigs. The belief that e-cigs can be an alternative to c-cigs and represent a quitting method, seems to have turned into ‘fact’, despite lack of evidence for e-cig safety and effectiveness for smoking cessation^5^. Preventing youth initiation and transition to established smoking are public health goals that bear great implications for the future^6^. An e-cig cartridge usually contains nicotine, a substance notorious for its malign influence on youths, mainly due to its deleterious long-term effects on adolescent brain development^7^. Another major negative consequence of the e-cig is that it leads to nicotine experimentation and addiction; e-cigs provide potential pathways for youth transferring to other forms of tobacco products.

Nevertheless, there has been some debate on the impact of first using e-cigs on future cigarette smoking. In some cross-sectional studies, ever e-cig users had higher odds for having smoking intention^8^ and openness to cigarette smoking than never users^9^. Trying e-cigs was a significant predictor of future cigarette smoking in many studies^10-14^. However, some studies using UK data reported that regular use of e-cigs is almost entirely concentrated in adolescents who already smoke and are not progressing to habitual use^15,16^. Aside from the increased risk of subsequent use of c-cigs and other illicit drugs, growing evidence indicates that e-cig use also exposes adolescents to several acute and long-term health risks^17^.

The characteristics of e-cig users have been already described. Those who are more likely to use e-cigs among US youth are generally senior students, Hispanics, Whites, and having lower levels of education^3^. In addition, some commonly cited reasons for using e-cigs are curiosity, flavoring/taste, and lack of awareness of the potential harms compared to other tobacco products^18^. Although the correlates of using e-cigs among the general population are well-known, there have been few studies to examine the characteristics of adolescents who started e-cigs before c-cig smoking. We hypothesized that the adolescents who chose e-cigs first, between c-cigs and e-cigs, have their own unique characteristics different from those of youths who chose c-cigs first. Given the recent surge in e-cig use among youth in the US, it is important to identify these characteristics. To achieve this aim, we examined the nationally representative data from the 2015 Korea Youth Risk Behavior Web-based Survey (KYRBWS).

## METHODS

### Participants

The KYRBWS is an anonymous, internet-based, self-reported questionnaire administered in classrooms to a nationally representative cross-section of middle school and high school students^18^. Participation was optional and those who completed the questionnaire were given a small gift. The Korea Centers for Disease Control and Prevention (KCDC) designed the questionnaire to be conducted yearly, since 2005, with the publicly available dataset to assess the prevalence of 7th to 12th grade students’ self-rated health risk behaviours. Data were collected from the 2015 KYRBWS study population (n=68043), consisting of boys and girls aged 12 to 18 years. Those who did not answer the questions about the timing of first use of e-cigs and c-cigs were excluded (n=83); finally we recruited 67960 adolescents. In the 2015 KYRBWS, the response rate was 97%. Details about the survey design and sampling methods are described elsewhere^19^ and are available at http://yhs.cdc.go.kr, including sampling weights for all Korean adolescents.

### Variables

Questions in KYRBWS about smoking included: ‘Have you ever used e-cigs?’ (yes/no), and ‘Have you used e-cigs in the past 30 days?’ (yes/no). Cigarette smoking questions included: ‘Have you ever smoked even one puff in your lifetime?’ (yes/no), and ‘How many days did you smoke, even one puff, in the past 30 days?’ (none/1–2 days/3–5 days/6–9 days/10–19 days/20–29 days/everyday). We defined ‘former smoker’ as a participant that ever smoked even one puff, but had not smoked in the past 30 days. Timing of first use of e-cigs and c-cigs was asked by school year. According to each response for e-cig and c-cig start school year, those who tried e-cigs earlier than c-cigs were classified as early e-cig users; while those who tried c-cigs earlier than e-cigs were classified as early c-cig users. If each response was the same year, we assumed that the responder tried both e-cigs and c-cigs in the same year. We finally classified subjects into six groups according to their timing of first using e-cigs or c-cigs; never user (neither tried e-cigs nor c-cigs), early e-cig user (tried e-cigs earlier than c-cigs), e-cig only (tried e-cigs only), same year (tried both in the same year), early c-cig user (tried c-cigs earlier than e-cigs), and c-cig only (tried c-cigs only). Questions were also asked about presence of friends or family members who currently smoke.

### Predictor variables

We have included variables related to starting smoking and vaping in previous studies. Sociodemographic factors included: age (school year), sex (male/female), residence (province/metropolitan city/other city), household income (mid-high/mid/low-mid), weekly allowance (<20/20–40/≥40 thousand KRW; exchange rate 1000 Korean Won about 0.834 US$), self-rated academic success (mid-high/mid/low-mid). As economic status and self-rated academic success were asked by 5 choices (‘high/mid-high/mid/low-mid/low’), high and mid-high groups were combined in ‘mid-high’; whereas low-mid and low groups were combined in ‘low-mid’. Questions about mental health, subjective health, and lifestyles included: ‘How much do you usually feel stress?’ (high/mild/little), ‘In the past 12 months, have you ever felt sad or hopeless to cause impaired daily life for more than two weeks?’ (yes/no), ‘How do you usually feel about your health?’ (good/moderate/poor), ‘How do you usually think of your happiness?’ (good/moderate/bad), ‘Have you ever drunk a high-caffeinated beverage during the past 7 days?’ (yes/no), ‘Have you ever used internet for recreation during weekday?’ (yes/no), ‘How do you think about your body shape?’ (slim/moderate/obese), and ‘Have you ever made an effort to control your weight during past 30 days?’ (no/losing/gaining/maintaining). Body mass index (BMI) was calculated by weight (kg) divided by the square of height (m^2^). Obesity was defined as a BMI of 25 kg/m^2^ or higher. KYRBWS included CRAFFT as screening test for problem drinking, composed of six items (Car, Relax, Alone, Forget, Friends, and Trouble)^20,21^.

A total score of 2 or higher is a positive screen, indicating a need for additional assessment.

Physical exercise was categorized as whether it took place for more than 5 days a week (<5 days vs ≥5 days).

### Statistics

The analysis used weighted values of strata samples and primary sampling units as provided in the public use dataset to compute descriptive statistics and logistics regression to account for the complex survey design. Prevalence estimates and standard errors were computed for the distribution of adolescents according to the timing of first using each tobacco product (never user/e-cig only/early e-cig user/same year/early c-cig user/c-cig only) by school year.

Bivariate analyses were employed to examine correlates between early e-cig and c-cig use. We included sociodemographic factors (school year, sex, residence, self-rated academic success, household income, and weekly allowance), mental health factors (feeling sadness, happiness, self-rated health and stress, obesity, self-assessed body shape, effort to reduce body weight), behavioral and lifestyle factors (alcohol abuse by CRAFFT, weekday internet use, having a caffeine drink, and regular moderate-intensity physical activity), and their smoking-related environments (exposure to secondhand smoke [SHS] at home, smoking friends and family members). Multivariate analysis was performed with a multivariate logistic regression model with every potential risk factor or marker with a score test inclusion criterion of p<0.05. Adjusted odds ratios (AOR) were estimated with their 95% confidence intervals (CIs). All analyses were conducted using Stata version 14.0 (Stata Corp, College Station, TX).

In this study, ethical approval is not required as the KYRBWS survey data are publicly available. All the participants signed an informed consent form.

## RESULTS

### Sample characteristics of study population

Table 1 shows the sample characteristics of the study population. Number of males were higher than females (35152 vs 32808). About half of students answered that their economic status (self-rated household income) belonged to middle class (46.8%) and most of them (94.0%) answered that they had no alcohol drinking problem according to CRAFFT, an alcohol abuse screening test. About 1 in 10 adolescents (11.9%) had a caffeine drink in the past 7 days. About 1 in 4 adolescents (23.5%) felt sad or hopeless in the past 12 months and about 1 in 6 adolescents (15.7%) responded that they had more than 60 minutes of moderate-intensity physical activity five times or more per week. Current and former e-cig users were 3.9% and 6.1%, respectively. The proportion of current c-cig users was higher than that of current e-cig users (7.7% vs 3.9%) and the proportion of ever c-cig users was also higher than that of ever e-cig users (17.3% vs 10.0%).

**Table 1:** General characteristics of study population (n=67960, N=3.35 M)

*Variables*	*Categories*	*n*	*wt% (95% CI)*
Sex	Male	35152	52.1 (51.7–52.5)
	Female	32808	47.9 (47.5–48.3)
School year	7	10770	13.7 (13.5–14.0)
	8	11419	15.6 (15.3–15.9)
	9	12045	17.6 (17.3–18.0)
	10	11111	17.4 (17.1–17.7)
	11	11106	17.6 (17.2–17.9)
	12	11509	18.1 (17.8–18.4)
Residence	Province	5649	6.3 (6.1–6.5)
	Metropolitan	29996	43.5 (43.1–43.9)
	Other city	32315	50.1 (49.7–50.6)
Household income	Low to mid	11529	16.9 (16.5–17.2)
	Mid	31934	46.8 (46.4–47.2)
	Mid to high	24497	36.4 (36.0–36.8)
Smoking status	Never	56397	82.7 (82.3–83.0)
	Former	6479	9.6 (9.4–9.9)
	Current	5084	7.7 (7.5–7.9)
Vaping status	Never	61362	90.0 (89.7–90.2)
	Former	4063	6.1 (5.9–6.3)
	Current	2535	3.9 (3.8–4.1)
Alcohol abuse (CRAFFT)	No	63997	94.0 (93.8–94.2)
	Yes	3963	6.0 (5.8–6.2)
Regular moderate-intensity physical activity (days/week)	<5	56933	85.8 (85.5–86.1)
	≥5	11027	14.6 (13.9–14.5)

n: unweighted sample size; wt%: weighted percentages with 95% confidence intervals in brackets. N: weighted sample size in million. CRAFFT: Car, Relax, Alone, Forget, Friends, Trouble.

### Timing of first using e-cigs or c-cigs by school year

**Figure 1:**
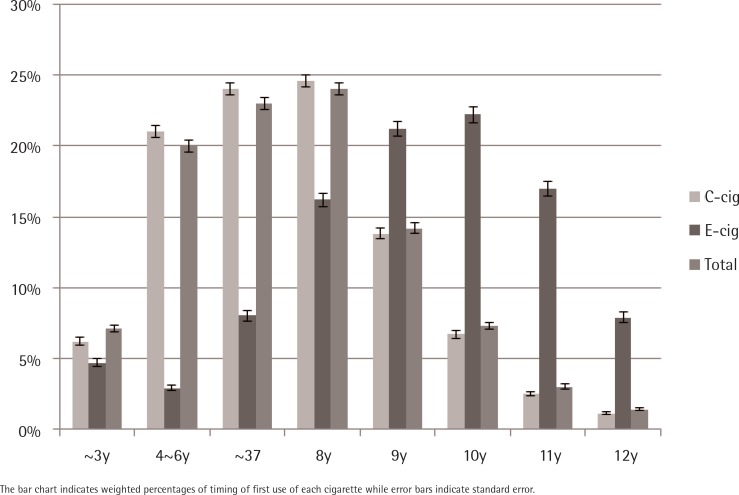
Timing of first use of electronic cigarette (E-cig) (n=6611) and conventional cigarette (C-cig) (n=11563) and any type cigarette (Total)(n=12428) by school year

Among adolescents included in the present study, only 1.7% tried e-cigs prior to c-cigs (Table 2). The same proportion responded that they started both in the same year and 15.2% of the total sample tried c-cigs earlier than e-cigs, while 8.7% of adolescents had experience of both c-cigs and e-cigs. Figure 1 shows that the timing of first using c-cigs tended to be earlier than that of e-cigs. The peak school year of first using c-cigs was the 8th, but that of e-cigs was the 10th year. Among adolescents who ever used any type of cigarette, the peak school year of first using any type of cigarette was the 8th year.

**Table 2:** Weighted prevalence^a^ of either electronic cigarette user and/or conventional cigarette user according to the timing of first using each type of cigarette and school year (n=67960, N=3.35 M)

*School Year*	*Never*	*Early E-cig user*	*E-cig only user*	*Same year*	*Early C-cig user*	*C-cig only user*	*Early E-cig user ratio^b^*
	*n=55532*	*n=241*	*n=865*	*n=1151*	*n=4340*	*n=5831*	
7	95.3 (0.2)	0.1 (0.0)	0.7 (0.1)	0.5 (0.1)	0.5 (0.1)	2.9 (0.2)	17.0 (0.8/4.7)
8	89.1 (0.3)	0.3 (0.1)	1.0 (0.1)	2.2 (0.2)	2.0 (0.1)	5.3 (0.2)	11.9 (1.3/10.9)
9	84.1 (0.4)	0.4 (0.1)	1.6 (0.1)	2.1 (0.1)	4.4 (0.2)	7.4 (0.3)	12.6 (2.0/15.9)
10	77.6 (0.4)	0.4 (0.1)	1.6 (0.1)	2.3 (0.2)	7.8 (0.3)	10.2 (0.3)	8.9 (2.0/22.4)
11	73.6 (0.5)	0.4 (0.1)	1.5 (0.1)	1.6 (0.1)	11.1 (0.3)	11.9 (0.3)	7.2 (1.9/26.4)
12	72.6 (0.5)	0.5 (0.1)	1.3 (0.1)	1.4 (0.1)	11.8 (0.3)	12.4 (0.3)	6.6 (1.8/27.4)
Total	81.3 (0.2)	0.4 (0.0)	1.3 (0.0)	1.7 (0.1)	6.6 (0.1)	8.6 (0.1)	9.1 (1.7/18.7)

n: unweighted sample size. N: weighted sample size in million. E-cig: electronic cigarette. C-cig: conventional cigarette.

a Weighted percentages with standard errors in brackets.

b Ratio of early E-cig user among any tobacco product user, numbers in per cent.

### Comparison of characteristics of initial e-cig user with initial c-cig user

Table 3 presents the differences of covariates between initial e-cig user and initial c-cig user. Apart from being male and younger, in general, those who adopted a healthy lifestyle and were in better condition, except for use of caffeine beverages, tended to start e-cigs earlier than c-cigs. More family income (8.6% vs 11.7%, p<0.001), better school scores (9.0% vs 11.0%, p=0.013), more regular physical activity (9.3% vs 11.7%, p=0.001), less stress (9.0% vs 11.3%, p=0.011), better feeling of health and happiness (8.5% vs 10.5%, p=0.001; 9.8% vs 10.6%, p=0.003, respectively), less use of weekday internet for recreational purpose (8.7% vs 11.5%, p<0.001) were associated with using first e-cigs before c-cigs. Interestingly, having a caffeine drink (9.2% vs 12.9%, p<0.001) and exposure to SHS (8.2% vs 12.2%, p<0.001) were associated with earlier use of e-cigs. No significant differences of residence, weekly allowances, obesity, effort to reduce body weight, subjective cognition of body shape, feeling sadness, and having family members who smoked were found.

**Table 3:** Comparison (%) of sociodemographic, lifestyle, mental health-related factors between early conventional cigarette and electronic cigarette attempt (n=12428)

*Factors*	*Variables*	*Categories*	*Early C-cig user*	*Early E-cig user*	p*
			*n=10171*	*n=1106*	
Sociodemographic factors	Sex	Male	89.7	10.3	0.004
		Female	91.6	8.4	
	School year	7	83.3	16.7	<0.001
		8	85.3	14.7	
		9	85.5	14.5	
		10	90.2	9.8	
		11	92.6	7.4	
		12	93.3	6.7	
	Residence	Province	90.0	10.0	0.565
		Metropolitan	90.6	9.5	
		Other city	89.9	10.1	
	Household income	Low-mid	91.4	8.6	<0.001
		Mid	90.8	9.2	
		Mid-high	88.3	11.7	
	Weekly allowance (thousand KRW)	<20	89.8	10.2	0.547
		20–40	90.4	9.6	
		≥40	90.2	9.5	
	Self-rated academic success	Low-mid	91.0	9.0	0.013
		Mid	90.0	10.0	
		Mid-high	89.0	11.0	
Mental health factors	Obesity (BMI >25 kg/m^2^)	No	90.8	9.2	0.868
		Yes	91.0	9.0	
	Effort to reduce body weight	No	90.1	9.9	0.504
		Yes	90.5	9.5	
	Subjective cognition of body shape	Not obese	89.8	10.2	0.055
		Obese	90.9	9.1	
	Stress	High	91.0	9.0	0.011
		Mild	90.1	9.9	
		Little	88.6	11.3	
	Sadness	No	90.5	9.5	0.103
		Yes	89.5	10.5	
	Subjective health	Good	89.5	10.5	0.001
		Moderate	92.0	8.0	
		Bad	91.6	8.5	
	Subjective happiness	Good	89.4	10.6	0.003
		Moderate	91.2	8.8	
		Bad	90.2	9.8	
Smoking-related environments	SHS exposure	No	91.8	8.2	<0.001
		Yes	87.8	12.2	
	Presence of family members who smoked	No	90.5	9.5	0.381
		Yes	90.0	10.0	
	Presence of friends who smoked	No	88.0	12.1	<0.001
		Yes	90.7	9.3	
Behavioral & Lifestyle factors	Alcohol abuse (CRAFFT)	No	89.0	11.0	<0.001
		Yes	94.1	5.9	
	Use of caffeine drink	No	90.8	9.2	<0.001
		Yes	87.1	12.9	
	Weekday internet use for recreation	No	88.5	11.5	<0.001
		Yes	91.3	8.7	
	Regular moderate-intensity physical activity (days/week)	<5	90.7	9.3	0.001
		≥5	88.3	11.7	

C-cig: conventional cigarette. E-cig: electronic cigarette. Thousand KRW: 1000 Korean Won, about 0.834 US$. CRAFFT: Car, Relax, Alone, Forget, Friends, and Trouble. BMI: body mass index. SHS: secondhand smoke.

* By chi-squared test.

### Correlates of first using e-cigs prior to c-cigs

As mentioned in the data analysis section, we employed multivariate logistic regression model to find characteristics to be associated with first using e-cigs prior to c-cigs among users of either e-cigs or c-cigs not in the same year (n=12428) (Table 4). The general characteristics of e-cig users among the whole sample (n=67960), which used the same factors in the model, are presented for comparison in Supplementary Table 1.

**Table 4:** Multivariate analysis of early electronic cigarette user among adolescents who ever tried either electronic cigarettes or conventional cigarettes not in the same year (n=12428)

*Variables*		*AOR*	*95% CI*	*p*
Sex	Male vs female	1.20	1.03–1.42	0.022
School year	7	2.23	1.66–2.99	<0.001
	8	1.98	1.57–2.50	<0.001
	9	2.08	1.69–2.55	<0.001
	10	1.40	1.14–1.71	0.001
	11	1.07	0.88–1.32	0.484
	12	Ref.		
Household income	Low to mid	Ref.		
	Mid	1.01	0.86–1.20	0.869
	Mid to high	1.21	1.01–1.45	0.034
Self-rated academic success	Low to mid	Ref.		
	Mid	1.13	0.97–1.33	0.124
	Mid to high	1.19	1.02–1.39	0.024
Stress	Much	Ref.		
	Mild	1.04	0.89–1.21	0.623
	Little	1.07	0.88–1.30	0.479
Subjective health	Good	Ref.		
	Moderate	0.85	0.71–1.01	0.074
	Bad	0.99	0.75–1.31	0.958
Subjective happiness	Good	Ref.		
	Moderate	0.85	0.81–1.01	0.057
	Bad	0.97	0.73–1.29	0.837
Exposure to SHS at home	Yes vs no	1.63	1.43–1.86	<0.001
Presence of friends who smoked	Yes vs no	0.92	0.78–1.08	0.319
Alcohol abuse (CRAFFT)	Yes vs no	0.57	0.48–0.68	<0.001
Use of caffeine drink	Yes vs no	1.44	1.24–1.68	<0.001
Weekday internet use for recreation	Yes vs no	0.69	0.60–0.78	<0.001
Regular moderate-intensity physical activity (days/week)	≥5 vs others	1.13	0.97–1.31	0.120

AOR: adjusted odds ratio. CI: confidence interval. CRAFFT: Car, Relax, Alone, Forget, Friends, and Trouble. BMI: body mass index. SHS: secondhand smoke.

In the fully-adjusted model, being initial e-cig user was significantly associated with male sex (AOR=1.20, 95% CI: 1.03–1.42). Compared to 12th grade, the younger students were likely to use e-cigs earlier (p-trend<0.01, not shown). Adolescents with higher family income (AOR=1.21, 95% CI: 1.01–1.45) and better school performance (AOR=1.19, 95% CI: 1.02–1.39) were associated with earlier use of e-cigs, but several mental health factors (stress, subjective health, and happiness) and regular physical activity were not significantly associated. Adolescents who had alcohol abuse and weekday use of internet for recreation were less likely to earlier use e-cigs, but having caffeine beverages was significantly associated with earlier use of e-cigs (AOR=1.44, 95% CI: 1.24–1.68). Exposure to SHS at home was associated with earlier use of e-cigs (AOR=1.63, 95% CI: 1.43–1.86), but presence of smoking friends was not (p=0.319).

## DISCUSSION

The study sample we analysed consisted of 10.0% of those who had previous experiences with e-cigs and 3.9% of the current e-cig users. Among the adolescent group with any previous e-cig experiences, the proportion of those who had ever used both types (dual-users) was 85.6%, and 80.5% for the current dual-users. This trend of the dual-user demography paralleled the findings of a previous Korean study based on the 2011 KYRBWS data^4^, and showed a clear correlation between the two studies. This study suggests that e-cig did not replace cigarette smoking or decrease the frequency of smoking, and thus, most e-cig users are dual-users, rather than only e-cig users who used e-cigs as an alternative to c-cigs. Although several studies also reported that regular use of e-cigs is almost entirely concentrated in adolescents who already smoke and are not progressing to habitual use^15,16^, the US survey revealed that e-cig users had surpassed cigarette smokers, especially among young adolescents^3^. This survey had also indicated that as younger students tend to adopt e-cigs earlier than c-cigs, knowledge of such characteristics is critical in understanding these changing trends. With the suggestion that vapers have a higher likelihood of progressing into a smoker in the future, a recent study presented a plausible explanation for the relationship between e-cigs and smoking implementation (common liability) by hypothesizing that smokers are also more likely to use e-cigs. Individual choices between e-cigs and c-cigs may show divergence according to environmental factors, particularly communal predisposition to nicotine^22^. In this context, this research sought to determine the prevalence of initial e-cig use over c-cig use among adolescents and examine the traits of those who had earlier direct contact with e-cigs compared to c-cigs.

Supplementary Table 1 displays distinct features of early e-cig users that differ from typical adolescent characteristics: distinct gender difference, reversed trend of family income, low school performance, and problem drinking. However, the two groups also bore similarities in that the rate of weekly internet use and the amount of caffeinated beverage consumed were parallel to each other. Interestingly, our study found that among those who experienced either e-cigs or c-cigs, adolescents who behaved more prudently tended to choose e-cigs over c-cigs in the initial stages. Besides, several risk factors such as mental health (depression and stress), obesity, and familial/peer smoking were not correlated with e-cig selections in multivariate analysis.

Several risk-involving behaviours such as alcohol, smoking and drug use, are known to be prevalent among adolescents. It was also discovered that the use of e-cigs was associated with problem alcohol drinking and smoking, although choosing e-cigs over c-cigs was associated with a less alcohol problem, higher school performance, and less use of weekday internet. Use of e-cigs, which is closely related to smoking, can be regarded as risky behaviour, on the other hand, participants who were recorded as choosing e-cig in their lifetime had less risky behaviour except for having caffeinated beverages. As caffeinated beverages are often consumed to improve concentration during study sessions, this result might be based on the pursuit of higher academic scores. However, e-cig initiation still is a valid public health concern, having significant social consequences such as the uptake of c-cig smoking (gateway theory). Therefore, raising awareness about e-cig initiation will be crucial to inhibit this social scourge.

A previous study showed that peer relationship among adolescents has a great influence on the initiation of c-cig and e-cig use^23^. We also found that peer smoking is associated with e-cig initiation in the total sample; however, there were no significant findings in terms of peer/familial smoking. Since the KYRBWS conducted a smoking-specific questionnaire based on peer/familial smoking and secondhand exposure to cigarettes, the extent of the effect of the surrounding e-cig users on e-cig initiation is difficult to ascertain.

This study suggests that the characteristics of adolescents who start e-cigs prior to c-cigs are significantly different from those who start with c-cigs. Currently, e-cigs have become relatively more accessible to teenagers than in the past, as the environmental pressures for smoking have increased. Therefore, it is crucial to improve school-based e-cig educational programs, not only for those who adopt risky behaviours but for all students, to raise awareness of e-cigs among the young. As the tobacco market and social smoking trends are constantly changing (including recent heated tobacco products), capitalizing on the global/national/subnational surveillance and monitoring system for newly-created tobacco products has become ever more vital, as suggested by WHO-FCTC (Framework Convention on Tobacco Control). Also, employing a more robust, pragmatic strategy at different levels (individual, family, schools, communities, and health service providers) and utilizing this information to establish a more comprehensive policy would further assist in tobacco control and protection of public health.

### Limitations

Our study has some limitations. First, as KYRBWS was performed in an internet-based, self-administered way, some responses obtained may be false, thus distorting the data. A small portion of the survey conducted was in the form of a cascade model, therefore there is a small possibility that such long-list questions might have led the respondents astray, mainly due to lack of interest and enthusiasm. However, the survey’s high response rate (over 95%) and respondent anonymity may serve to overcome these issues. Other limitations of this study are that the cross-sectional design of the sample obtained cannot be used to reveal causality and that some selection bias may have been applied as those who responded the ‘same year’ (1.7 % of the whole sample) were excluded from the overall sample.

## CONCLUSIONS

The proportion of adolescents who first started using e-cigs prior to c-cigs is less than 10% for any tobacco product. However, younger students are more likely to first start with e-cigs. Apart from being younger, participants were more likely to have healthier lifestyles and positive behaviours compared to those who started with c-cigs. Gender differences shown in the general characteristics of ever e-cig user were much attenuated with the timing of first use.
