# Supplementary material for: Prevalence and correlates of prior experimentation with e-cigarettes over conventional cigarettes among adolescents: Findings from the 2015 Korea Youth Risk Behaviour Web-based Survey
Source: Tob Prev Cessat. 2019 Oct 7;5:33. doi: 10.18332/tpc/112595 (PMC7205116; doi:10.18332/tpc/112595)
Supplement: Supplementary file 1 [file TPC-5-33-s1.pdf]

Supplementary Table 1. Correlates of ever-experimentation with electronic cigarette among whole sample (n=67960, N=3.35).

		aOR	95% CI	P
Gender	Male vs female	**2.63**	2.41-2.87	<0.001
School grade	7	ref		
	8	**1.54**	1.27-1.86	<0.001
	9	**1.72**	1.42-2.07	<0.001
	10	**1.54**	1.28-1.85	<0.001
	11	**1.46**	1.21-1.75	<0.001
	12	**1.36**	1.13-1.63	0.001
Self-rated household income	Low to mid	ref		
	Mid	0.95	0.87-1.04	0.393
	Mid to high	1.09	0.99-1.21	0.074
Self-rated academic success	Low to mid	ref		
	Mid	**0.80**	0.73-0.87	<0.001
	Mid to high	**0.65**	0.59-0.70	<0.001
Stress	Much	ref		
	Mild	**0.88**	0.73-0.87	0.003
	Little	**0.88**	0.79-0.98	0.023
Subjective health	Good	ref		
	Moderate	**0.87**	0.79-0.95	0.002
	Bad	0.91	0.79-1.06	0.227
Subjective happiness	Good	ref		
	Moderate	1.06	0.97-1.15	0.206
	Bad	1.02	0.90-1.16	0.729
Experience of cigarette smoking	Ever vs never	**24.89**	22.9-27.0	<0.001

Exposure to SHS at home	Yes vs no	**1.37**	1.27-1.47	<0.001
Presence of friends who smoked	Yes vs no	**3.92**	3.54-4.34	<0.001
Alcohol abuse (CRAFFT)	Yes vs no	**3.44**	3.13-3.78	<0.001
Use of caffeine drink	Yes vs no	**1.32**	1.20-1.44	<0.001
Weekday internet use for recreation	Yes vs no	**0.78**	0.73-0.84	<0.001
Regular moderate-intensity physical activity	>=5days/wk vs others	**1.21**	1.11-1.32	<0.001

aOR, adjusted odds ratio; CI, confidence interval; KW, kilo-WON (₩); CRAFFT, acronym of Car, Relax, Alone, Forget, Friends, and Trouble; BMI, body mass index; SHS, secondhand smoke

n, unweighted sample size; weighted percentages with standard errors in bracket
N: Weighted sample size in millions

© 2019 Hyeon J. H.
